# Serum antioxidant micronutrient levels in pre-eclamptic pregnant women in Enugu, south-East Nigeria: a comparative cross-sectional analytical study

**DOI:** 10.1186/s12884-020-03081-w

**Published:** 2020-07-06

**Authors:** Joseph Tochukwu Enebe, Cyril Chukwudi Dim, Emmanuel Onyebuchi Ugwu, Nympha Onyinye Enebe, Ijeoma Angela Meka, Kingsley Chukwu Obioha, George Uchenna Eleje, Uchenna Ifeanyi Nwagha

**Affiliations:** 1 Department of Obstetrics & Gynaecoclogy, Enugu State University of Science and Technology College of Medicine/Teaching Hospital, Parklane, P.M.B. 1030, Enugu, 400001 Nigeria; 2 Departments of Obstetrics and Gynaecology, Faculty of Medical Sciences, College of Medicine, University of Nigeria, Ituku-Ozalla, Enugu, Nigeria; 3 Institute of Maternal and Child Health, College of Medicine, University of Nigeria, Ituku-Ozalla, Enugu, Nigeria; 4 Departments of Obstetrics and Gynaecology, University of Nigeria Teaching Hospital (UNTH), Ituku-Ozalla, Enugu, Nigeria; 5 Department of Community Medicine, UNTH, Ituku-Ozalla, Enugu, Nigeria; 6 Department of Chemical Pathology, Faculty of Medical Sciences, College of Medicine, University of Nigeria, Ituku-Ozalla Campus, Enugu, Nigeria; 7 Effective Care Research Unit, Departments of Obstetrics and Gynaecology, Faculty of Medicine, Nnamdi Azikiwe University, Nnewi Campus, Nnewi, Nigeria; 8 Department of Physiology, Faculty of Basic Medical Sciences, College of Medicine, University of Nigeria, Enugu Campus, Nsukka, Nigeria

**Keywords:** Pre-eclampsia, Micronutrients, Serum, Antioxidants

## Abstract

**Background:**

Pre-eclampsia is a common obstetric complication of pregnancy in Nigeria, and oxidative stress has been implicated in its aetiopathogenesis. Despite this fact, there is a paucity of information regarding the serum antioxidant micronutrient status of pre-eclamptic Nigerian women. The objective of the was to determine the mean serum levels of some antioxidant trace elements (copper, zinc, selenium, magnesium, manganese) in pre-eclamptic pregnant women and compare with that of healthy pregnant women in Enugu, South-Eastern, Nigeria.

**Methods:**

A cross-sectional analytical study was carried out at the Obstetrics and Gynaecology department of the University of Nigeria, Teaching Hospital Ituku-Ozalla, Enugu. Using atomic absorption spectrophotometry, the sera of 81 pregnant pre-eclamptic and 81 matched healthy pregnant controls were analyzed for the antioxidant micronutrients. Both descriptive and inferential analysis was performed using the statistical package for social sciences (SPSS) version 21.0 and a *P* value of < 0.05 was considered to be statistically significant.

**Results:**

The mean serum levels of copper, selenium, and magnesium were found to be significantly lower in the pre-eclamptic pregnant group when compared to the healthy pregnant controls (*p* < 0.05). The mean serum levels of zinc and manganese did not differ between the two groups (*p* > 0.05). All the mean serum levels of micro-nutrients studied did not vary by category of pre-eclampsia (with or without severity findings) except manganese which was significantly lower in pre-eclamptic women without severity findings when compared to those with severity findings (*p* = 0.043).

**Conclusions:**

The serum levels of copper, selenium, and magnesium were significantly lower among pre-eclamptics when compared to their normal healthy controls. Low levels of selenium, copper, and magnesium may have contributed to the incidence of pre-eclampsia in our environment.

## Background

Pre-eclampsia is the onset of hypertension accompanied with proteinuria and/or evidence of maternal acute kidney injury (AKI), liver dysfunction, neurological features, haemolysis or thrombocytopaenia, or fetal growth restriction after mid-pregnancy in a previously normotensive woman, and this condition resolves completely by the sixth week after delivery [1, 2]. It is a transient systemic condition, that can be fatal in pregnancy. The condition has a rapid progression, and it is characterized by high blood pressure and occurrence of proteinuria classically and /or impaired liver function, low platelet, severe persistent right upper quadrant or epigastric pains, visual disturbances pulmonary oedema, and new-onset headache [2]. Pre-eclampsia among other medical complications of pregnancy is the most common but least understood and it is associated with increased maternal and perinatal morbidity and mortality [3–6]. It affects 5–7% of all pregnancies worldwide [6]. In low-income countries, pre-eclampsia has contributed to 20–80% of increased maternal mortality in those countries. In high-income countries, a five-fold rise in perinatal mortality accounting for 15% of preterm births has been observed [6]. It is one of the most common indications for elective delivery which serves as a treatment for the disease.

Notwithstanding the prevalence and severity of this disease, the aetiopathogenesis of pre-eclampsia has not been fully understood. Placental oxidative stress has been identified as a major pathway to the development of pre-eclampsia [7, 8]. Oxidative stress occurs when the intracellular production of reactive oxygen species (ROS) overwhelms the ability of systemic antioxidants to neutralize the systemic oxidants thereby preventing oxidative cellular injury. In pre-eclamptic women, it is observed that both the expression and activity of some vital antioxidant proteins are reduced in the substance of the placenta leading to the disequilibrium between pro-oxidants and antioxidants. Oxidative stress occurring in both the placenta and maternal tissues contribute to the observed multi-systemic effect and the generalized maternal systemic inflammatory activation [9, 10]. Excessive generation of ROS leads to repeated and progressive placental ischaemia-reperfusion injury and continuous release of placental factors that mediate inflammatory responses [11]. However, the placenta is naturally protected by some antioxidant defenses which include the selenium-dependent glutathione peroxidases, thioredoxin reductases, selenoprotein-P, and copper/zinc and manganese superoxide dismutases (Cu/Zn and Mn SODs). These antioxidant enzymes defense systems help to prevent the placenta from undue harm. This is the basis of the involvement of micronutrients and other antioxidants in the aetiopathogenesis of pre-ecplampsia.

Copper is a component of some enzymes and serves as a co-factor to superoxide dismutase, which is an antioxidant [12]. The action of copper in redox actions and the formation of free radicals therefore signifies its importance in the pathogenesis of pre-eclampsia. Similarly, selenium is a component of some vital enzymes that play roles in the antioxidant activities, so if serum values of these enzymes are low; it can result in numerous pathways that can lead to pre-eclampsia. Likewise, magnesium is an important cofactor to some endogenous antioxidant enzymes [13] and the usefulness of magnesium sulfate in the treatment of eclamptic seizures [14] suggests that low serum of the element may be associated with the development of pre-eclampsia and eclampsia.

The severity and high prevalence of pre-eclampsia in low-income countries have made researchers strongly believe in the role of nutrition especially trace elements in the aetiopathogenesis of pre-eclampsia [5, 15, 16]. The epidemiology of pre-eclampsia showed that the disease is more prevalent among indigent women, and this has further strengthened the possibility that nutrients may be involved in this disorder [17, 18]. Previous studies on the relationship between the plasma concentrations of trace elements and pre-eclampsia have produced reports which had shown various levels of significance of antioxidant micronutrients in this group of women [19–23].

Although some studies have linked low serum concentrations of the above-named trace elements to the pathogenesis of pre-eclampsia [20, 21, 23], other reports have shown no relationship [19, 22], thus the need for further studies in this area.

This study may contribute globally to the existing unresolved controversies on the role of trace elements in the pathogenesis of pre-eclampsia, especially in low-income countries like Nigeria where these micronutrients are deficient in the local diet [24]. It may also provide baseline values for serum levels of some of the studied trace elements among pregnant women in Enugu, South-eastern Nigeria. Further information on the need for supplementation of these trace elements among our pregnant women may equally be obtained. Given the above, this study aimed to compare the maternal serum levels of copper, zinc, manganese, selenium, and magnesium in pre-eclamptic women with normal pregnancy.

## Methods

This study was carried out at the antenatal clinic, wards, and labour room at the University of Nigeria Teaching Hospital (UNTH), Ituku-Ozalla, Enugu, Nigeria. The hospital offers tertiary health services to patients from Enugu state and other parts of the southeastern region of Nigeria. It has about 800 beds and more than 5000 various health care workers [25]. UNTH is one of the major referral tertiary health care centers in southeastern Nigeria. The analysis of blood samples for the maternal serum levels of copper, zinc, manganese, selenium, and magnesium was however carried out at the Department of Chemical Engineering, Caritas University, Amorji-Nike, Enugu, Nigeria.

### Study area

Enugu State also called The Coal City State is located in the South-eastern part of Nigeria. The capital city is Enugu. It spreads towards the middle belt region of Nigeria and covers a land area of approximately 8727.1km^2^ [26]. The major occupation of the state is subsistence farming and animal rearing in rural areas and civil service in urban centers. The predominant tribe is Igbo and the population is about 3.26million [26] Enugu state has a crude birth rate of 45 per 1000, a crude death rate of 18 per 1000 of the population and the life expectancy of 51 years [27]. The maternal mortality ranges from 750 to 850 per 100,000 live births [28, 29].

Enugu metropolis is located in the tropical rain forest about 230 m above sea level. This tropical rain forest has gradually changed to guinea savannah due to deforestation and farming activities. The average annual rainfall is 1520-2030 mm and temperature ranges between 23.1 and 31 °C. The state has two major seasons, the rainy season (April to October) and dry season (November to February) with harmattan with dusty haze from the Sahara region between December and early February. The Enugu town has about 464,514 inhabitants [26]. The staple food in Enugu includes cassava, yam, cocoyam, beans, rice, egusi (melon), ogbono (Irvingia, African mango, bush mango), oha (African Rosewood leaves, Pterocarpus mildraedii) and other vegetables.

The mean micronutrient contents of River Nyaba and its tributary which drains most water from Enugu during both dry and rainy seasons are as follows: Copper (World Health Organization [WHO] permissible limit-2.0 mg/L, dry season-18.26 μg/L, wet season-13.47 μg/L), manganese (WHO limit-0.05 mg/L, dry season-1425.9 μg/L, wet season-3353.8 μg/L), zinc (WHO limit-3 mg/L, dry season-93.23 μg/L, wet season-253.82 μg/L), magnesium (WHO limit-100 mg/L, dry season-7.19 mg/L, wet season-6.8 mg/L) [30].

### Participants

The study participants were pre-eclamptic and healthy pregnant women attending the antenatal and booking clinics of the University of Nigeria Teaching Hospital, Ituku-Ozalla, Enugu, Nigeria. They also included the unbooked pre-eclamptic pregnant women admitted into antenatal, special care, and labour wards of the hospital.

#### Exclusion criteria

Pregnant women with other chronic illnesses such as human immunodeficiency virus (HIV) infection, diabetes mellitus, malignancy, and tuberculosis were excluded from the study. Also excluded were those on medications that could affect the levels of these trace elements in the serum e.g. magnesium sulfate, supplements containing these micronutrients, and pregnant women who smoked cigarettes and drank alcoholic beverages. Also, pregnant women who incidentally became pregnant while on copper-containing contraceptive devices, those whose gestational ages could not be ascertained and women with multiple pregnancies were also excluded.

### Study design and sample selection

The study was cross-sectional and analytical in design. The study group consisted of women (81) with pre-eclampsia who met the above study criteria while the control group consisted of matched healthy pregnant women (81) receiving antenatal care at the antenatal and booking clinics of the hospital. The sample size was calculated to be 83 for each group using the formula [31]: n = (Z_1_ + Z_2_)^2^ × 2(S)^2^ / (μ_2_-μ_1_)^2^ and attrition rate of 10%. Z_1_ was 1.96(95% CI), Z_2_ was 0.84%, and the mean standard deviation of mean serum level of copper was 1.9 μmol/l and a power of 80% [23].

The women in the study group were consecutively recruited after details of the study were made known to them and written informed consent obtained. The women in the control group were matched on a 1:1 ratio with those in the study group in terms of age, gestational age, parity, and body mass index. After identifying a consented and eligible participant in the study group, the next antenatal clinic attendee with normal pregnancy who matched was selected. For the unbooked pregnant pre-eclamptic women, they were recruited first at presentation before they were matched with the healthy pregnant women at the antenatal clinic.

The socio-economic status of the women was determined using the Olusanya criteria [32]. The social classes were grouped into six groups (1-1 V) with 1 being the highest class and V1 the lowest class. The social class of each participant was determined by the sum of scores of husband occupation and the wife’s educational level as described in the criteria.

Pre-eclampsia was defined in this study as “the occurrence of de novo hypertension (≥140/90mmHg) after 20 weeks’ gestation accompanied by proteinuria and/or evidence of maternal acute kidney injury (AKI), liver dysfunction, neurological features, haemolysis or thrombocytopaenia, or fetal growth restriction” [2]. For the study, two blood pressure values were taken 4 h apart and the average was taken as the participant’s blood pressure. Blood pressure measurement was taken with the women in sitting position using Accoson Mercury sphygmomanometer after at least 10 min of rest. Systolic blood pressure recorded at the appearance of sounds and diastolic blood pressure recorded at the muffling of sounds of Korotkoff (Korotkoff iv). Proteinuria exists if urinary protein concentration of ≥30 mg/dl (≥2+ on dipstick) in a minimum of two random urine samples collected 4 h apart or ≥ 300 mg of protein in 24-h urine [33, 34].

The severity of pre-eclampsia was categorized into pre-eclampsis without severity findings (mild) and with severity findings (severe) as follows [2]:.

#### Mild Pre-eclampsia currently regarded as pre-eclampsia without severity findings

blood pressure of ≥140–159/90-109 mmHg with two measurements taken 4 h apart and proteinuria of 2+ on the dipstick in a minimum of two random urine samples collected at least 4–6 h apart or 0.3 g or more of protein in 24-h urine but not up to 5 g [33, 34] with no severity findings as listed below.

#### Severe pre-eclampsia currently regarded as pre-eclampsia with severity findings

blood pressure of ≥160/110 mmHg or higher with proteinuria of 5 g or more in 24 h or 3+ of proteinuria on two random urine samples collected at least 4–6 h apart. Also, the diagnosis was made in the presence of any of these severity features: pulmonary oedema or cyanosis, oliguria (< 400 ml of urine in 24 h) or renal failure, epigastric pain and/or impaired liver function test or right hypochondrial pains, thrombocytopaenia, oligohydramnios, decreased fetal growth syndrome (Haemolysis, Elevated Liver enzymes, and low platelets) and maternal neurological disturbances (phosphene signals, diffuse tendon reflexes, tinnitus, eclampsia, etc) or persistent headaches (intrauterine growth restriction), placental abruption, and HELLP syndrome [33, 34].

#### Ethical approval and data collection

Data collection commenced after ethical approval by the University of Nigeria Teaching Hospital, Ituku-Ozalla Health Research Ethics Committee, and the National Postgraduate Medical College of Nigeria. The ethical certificate number was NHRE/05/01/2008B-FWA00002458-1RB00002323 and sample collection was performed between July 2013 and July 2016.

Consent was obtained from participants after adequate individualized counseling. Women who were not stable enough to give consent had their consent obtained by proxy from their closest relative. Five ml of blood was collected into a plain labelled specimen bottle and left at room temperature to clot. The serum was obtained by centrifugation for 10 min at 4000 rpm and stored at -20 °C until analysis.

### Biochemical analysis of the serum specimens

The levels of selenium, zinc, magnesium, copper, and manganese were estimated using atomic absorption spectrophotometry (AAS) [22, 23] The laboratory scientist was blinded on the study objectives and patient groups analyzed in the samples. Quality control was ensured using Commercially prepared quality control samples.

### Statistical analysis

The analysis was carried out with Statistical Package for Social Sciences (SPSS) version 21.0. Proportions were compared with Pearson Chi-square for categorical variables while means were compared using Students t test. All continuous variables were subjected to test for normality and data that were not normally distributed were corrected with Mann-Whitney U test. The mean values were represented as mean ± SD. Data are presented using tables as appropriate. Values were set at 95% confidence level, a *P*-value of less than 0.05 was considered significant.

## Results

### Basic characteristics of the study and control groups

The mean age of the participants in the study and control groups was 29.53 ± 5.38 and 29.31 ± 5.22 years respectively and age range was 18–40 years. Most of the participants belonged to the 30–34 years (29.6%) and 25–29 years (28.4%) age groups. Very few participants were recruited at the extremes of the reproductive ages; thus, only one participant (1.2%) per group for age group of 40 years and above and 2 participants (2.5%) per group for < 20 years age group. Most of the participants belonged to the class II and III socio-economic class for both the study (38.3, 32.1%) and control (46.9, 28.4%) groups respectively.

In addition, most of the participants in both groups were mainly nulliparous (35.8%) and multiparous (32.1%) women. The mean systolic blood pressure for the study and control groups was 174mmhg and 107 mmHg respectively while the mean diastolic blood pressure was 114 mmHg and 66 mmHg respectively. Details of the socio-demographic and anthropometric characteristics of the participants are shown in Table 1 below.

**Table 1:** Basic Characteristics of Study Participants

Variables	Variable sub groups (years)	Pre-eclampsia (*n* = 81)		Normal (*n* = 81)		P -value
		Frequency	%	Frequency	%	
Age groups	< 20	2	2.5	2	2.5	1.0
	20–24	16	19.8	16	19.8	
	25–29	23	28.4	23	28.4	
	30–34	24	29.6	24	29.6	
	35–39	15	18.5	15	18.5	
	≥40	1	1.2	1	1.2	
	Mean	29.53 ± 5.38		29.31 ± 5.22		
Educational Level	Primary	3	3.7	3	3.7	
	Secondary	41	50.6	25	30.9	0.036
	Tertiary	37	45.7	53	65.4	
	No formal education	0	0.0	0	0.0	
Social class	I	3	3.7	8	9.9	0.207
	II	31	38.3	38	46.9	
	III	26	32.1	23	28.4	
	IV	19	23.5	10	12.3	
	V	2	2.5	2	2.5	
Marital status	Single	6	7.4	0	0.0	0.028
	Married	75	92.6	80	98.8	
	Widowed	0	0.0	1	1.2	
Christian denomination	Catholic	50	61.7	49	60.5	0.792
	Anglican	9	11.1	7	8.6	
	Pentecostal	22	27.2	25	30.9	
Tribe	Igbo	77	95.1	71	87.7	0.079
	Hausa	0	0.0	0.0	0.0	
	Yoruba	1	1.2	0	0.0	
	Others	3	3.7	10	12.3	
Parity	Nulliparous(P_0_)	29	35.8	29	35.8	1.0
	Primiparous(P_1_)	22	27.2	22	27.2	
	Multiparous(P_2–4_)	26	32.1	26	32.1	
	Grandmultiparous(*P*_≥5_)	4	4.9	4	4.9	
BMI (kg/m^2^)	≤19.9	3	3.7	3	3.7	1.0
	20.0–24.9	17	21.0	17	21.0	
	25.0–29.9	20	24.7	20	24.7	
	30.0–34.9	23	28.4	23	28.4	
	35.0–39.9	8	9.9	8	9.9	
	≥40.0	10	12.3	10	12.3	
	Mean	30.29 ± 6.80		30.29 ± 6.80		

The values of serum copper, selenium, and manganese were significantly lower in pre-eclamptics when compared with the control group (*p* < 0.05). Serum levels of zinc and manganese did not vary between the two groups (*p* > 0.05). Table 2 below shows the details of the serum levels of the trace elements studied. The mean serum levels of Copper, Selenium, Zinc, and Magnesium in women with pre-eclampsia without severity and with severity findings did not differ significantly (p > 0.05). However, the mean serum levels of manganese in participants with pre-eclampsia without severity findings was significantly lower than that of women with severity findings (*p* = 0.006). The details of comparisons of the mean values of the micro-nutrients in participants with pre-eclampsia without and with severity findings are as shown in Table 3 below.

**Table 2:** Comparison of the mean serum level of micro-nutrients in pre-eclamptic pregnant women and controls

Trace Element	Pre-eclamptics (*n* = 81)	Controls (*n* = 81)	*p*-value
	Mean ± 2SD	Mean ± 2SD	
Copper (mg/l)	0.844 ± 0.57	1.670 ± 1.47	< 0.001
Selenium (mg/l)	0.842 ± 0.71	1.758 ± 3.35	0.017
Zinc (mg/l)	0.408 ± 0.39	0.535 ± 0.80	0.198
Magnesium (mg/l)	1.397 ± 0.98	5.044 ± 6.60	< 0.001
Manganese (mg/l)	0.0958 ± 0.11	0.1635 ± 0.34	0.088

**Table 3:** Comparison of the mean serum levels of micro-nutrients in participants with mild and severe pre-eclampsia

Trace elements	Pre-eclampsia without severity findings (*n* = 24)	Pre-eclampsia with severity findings (*n* = 57)	p - value
	Mean value (SD)	Mean value (SD)	
Copper(mg/l)	0.994 (0.519)	0.781 (0.586)	0.127
Zinc(mg/l)	0.300 (0.179)	0.453 (0.438)	0.105
Selenium (mg/l)	0.715 (0.743)	0.895 (0.698)	0.300
Manganese (mg/l)	0.0425 (0.071)	0.118 (0.122)	0.006
Magnesium(mg/l)	1.458 (0.889)	1.372 (1.022)	0.723

## Discussion

Determining the serum levels of trace elements/micro-nutrients (copper, zinc, selenium, magnesium, and manganese) provides insight into the understanding of the pathophysiology of pre-eclampsia [15, 35, 36]. In other to achieve this, the risk factors like age, gestational age, parity, and Body Mass Index (BMI) are associated with the development of hypertension and pre-eclampsia [37, 38] were controlled in this study. Therefore, the study and control groups were similar concerning their age groups, gestational age groups, BMI groups, and parity groups.

This study found that the mean serum levels of copper, selenium, and magnesium, were significantly lower in the pre-eclamptic women than the control, which is consistent with the results of similar studies [22, 23, 39–41]. Akinloye et al. [23] in their cross-sectional study in Osogbo, Nigeria reported significantly reduced plasma levels of copper in women with pre-eclampsia compared with healthy pregnant controls. Ugwuja et al. [22] in a similar study in Abakaliki, Nigeria also observed significantly low mean levels of copper in pre-eclamptic women compared to their healthy counterparts (6.02(±7.23) μmol/l versus 10.17(±9.84) μmol/l, *p* < 0.05). The last two studies which are in keeping with our result findings on serum copper were conducted in a similar environment and these participants consume similar food items and share a close genetic and socio-demographic background. Likewise, Rothore et al. [42] in a comparative cross-sectional study in India, studied 14 pre-eclamptic and 47 normal pregnant women and found that mean serum copper levels were significantly decreased in pre-eclamptic women compared to the healthy pregnant controls. However, this finding contrasts that of a similar study in Tehran, Iran [43] where serum copper did not differ between pre-eclamptic and normal pregnant women.

Furthermore, our study finding on serum selenium was similar to that of Neda et al. [44] who in a cohort study in Iran, found a significantly lower mean serum level of selenium in pre-eclamptics compared to the healthy pregnant women. In addition, in a similar cross-sectional study, Akinloye et al. [23] in Osogbo, Nigeria found that levels of selenium were significantly reduced in pre-eclamptics compared to matched healthy controls (0.6 ± 0.1 vs 1.3 ± 0.4 μmol/L). However, Rayman et al. [45] in a cross-sectional study in the UK analyzed serum samples of 19 pre-eclamptic women with matched healthy controls. They found no significant difference between the two groups though the serum levels were reduced in both groups. The small sample size in this study may have affected their findings. Selenium supplementation may be useful to women predisposed to pre-eclampsia as few placebo-controlled randomized control trials on selenium supplementation have reported lower rates of pre-eclampsia and /or pregnancy-induced hypertension [46, 47].

Nevertheless, the result of the mean serum level of magnesium obtained in our study differed from the result of a similar study in Elazig, Turkey [48] where serum magnesium did not vary significantly between the pre-eclamptic women and the normal pregnant controls. The small number of pre-eclamptic women (*n* = 30) used in that study might have affected the study’s precision. In contrast, a similar study in Beer-Sheva, Israel [49] noted that mean serum magnesium was significantly higher in pre-eclamptic pregnant women when compared to normal pregnant women. However, unlike in our study, the latter study involved women that had received magnesium sulfate before the blood samples for the serum trace elements were collected and this might explain the higher level of magnesium in their pre-eclamptic population. Furthermore, another study from Kerman province, Iran [50] found that there was no significant difference in the mean serum levels of calcium, zinc, and magnesium among normal, mild (pre-eclampsia without severity findings) and severe (pre-eclampsia without severity findings) pre-eclamptic women.

In our study, though the mean serum levels of Zinc and Manganese in the pre-eclamptic women were lower than those of their normal pregnant controls, the differences were not significant. This study finding is similar to that obtained from a study in Kerman province, Iran [51] and Shatin, Hong Kong [50] where the mean zinc and manganese levels were not significantly lower in pre-eclamptic women compared to normal pregnant women. Likewise, another study in Gorgan, Iran, equally recorded no significant difference in the serum zinc concentrations between pre-eclamptic and normal pregnant women [52]. The minor differences which may not have been eliminated in the above-highlighted studies that recorded significant differences in the serum levels of zinc and manganese in the two groups studied may be due to differences in age, BMI, gestational ages and parity groups which were controlled in our study.

The vital roles of manganese and zinc as components of enzymes with antioxidant activities in humans in the pathogenesis of pre-eclampsia are fully elucidated [20, 22, 23]. However, unlike in this study a meta-analysis [53] and two other studies from Lagos, Nigeria [54] and Harran, Turkey [55] found increased serum zinc concentration among pre-eclamptic women compared to normal pregnant controls. The latter studies [54, 55] used fasting blood samples, unlike our current study that utilized non-fasting samples, which may account for the differences observed. The varying population studied by the meta-analysis with differences in food and soil content of zinc might have been responsible for the result of the meta-analysis on zinc. Also, the differences in the nutritional content of food consumed by the participants in the study population may be responsible for the difference noted in this present study.

The pathogenesis of pre-eclampsia cuts across all systems in the body and the progression of this disease from mild to severe disease can sometimes not be easily predictable. Besides, some women with suspected mild (pre-eclampsia without severity findings) disease develop eclampsia even at normal blood pressures. This inability to determine the thin demarcation between the mild (pre-eclampsia without severity findings) and severe (pre-eclampsia with severity findings) pre-eclampsia in some pre-eclamptic women may be responsible for the result obtained in this study where there was no significant difference in the mean serum level of selenium, copper, zinc, and magnesium between women with mild (pre-eclampsia without severity findings) and severe (pre-eclampsia without severity findings) pre-eclampsia. This result is consistent with that of a similar study in Iran where there were no significant differences in the serum magnesium and zinc between the mild (pre-eclampsia without severity findings) and severe (pre-eclampsia with severity findings) pre-eclamptic women [51]. Also, there was no significant difference in serum zinc levels between the mild (pre-eclampsia without severity findings) and severe (pre-eclampsia without severity findings) pre-eclamptic women as documented in another similar study [56]. In contrast, other studies [57–60] noted significantly decreased serum levels of zinc, copper, and magnesium in women with severe (pre-eclampsia with severity findings) pre-eclampsia when compared to those with mild pre-eclampsia. The differences noted in the serum levels of these micronutrients between severe (pre-eclampsia with severity findings) and mild pre-eclampsia (pre-eclampsia without severity findings) in this study may also depend on the age ranges of the women used in the study, the nutritional status of the women and other differences in the soil content of these trace elements in the study area used. However, this finding may have been affected by the sample size of women with pre-eclampsia with severity findings and pre-eclampsia without severity findings. Since the sample size for this study was calculated to find a difference in the primary outcome measure not the severity of pre-eclampsia.

Furthermore, the marked significant difference in the serum level of manganese between the mild pre-eclampsia without severity findings) and severe pre-eclampsia (pre-eclampsia with severity findings) is noteworthy. It is possible that decreasing serum manganese level may be a marker of worsening severity of pre-eclampsia as observed with uric acid where increasing values suggests worsening disease condition [61]. Further studies with larger sample sizes are required in this regard.

The strengths of this study lie in the large sample size used. Also, this study analyzed as much as five trace elements that have not been done in the recent past in Nigeria. One of the limitations of the study is the use sample size for the comparison of mean between normal and pre-eclamptic women to compare participants with (severe pre-eclampsia) or without (mild pre-eclampsia) severity findings. Therefore, multicenter studies with appropriate sample sizes for comparison of severity levels are strongly recommended.

## Conclusion

This study demonstrated that the mean serum levels of copper, selenium, and magnesium were significantly lower in pre-eclamptic when compared to normal pregnant controls thus, the reduced serum levels of these elements might be associated with the pathogenesis of pre-eclampsia in the study environment. On the other hand, mild pre-eclamptics had significantly lower serum manganese when compared to severe pre-eclamptics thus, rising serum manganese might be a marker for worsening severity of pre-eclampsia in our environment.

## Data Availability

The data sets used and/or analysed during the current study are available from the corresponding author on reasonable request.

## Abbreviations

AAS: Atomic absorption spectrophotometry
BMI: Body Mass Index
Cu/Zn and Mn SODs: Copper/zinc and Manganese superoxide dismutases
HIV: Human immunodeficiency virus
ROS: Reactive oxygen species
SPSS: Statistical package for social sciences
UNTH: University of Nigeria Teaching Hospital
WHO: World Health Organization
